# Mineralization of Early Stage Carious Lesions *In Vitro*—A Quantitative Approach

**DOI:** 10.3390/dj3040111

**Published:** 2015-10-10

**Authors:** Hans Deyhle, Iwona Dziadowiec, Lucy Kind, Peter Thalmann, Georg Schulz, Bert Müller

**Affiliations:** 1 Biomaterials Science Center, University of Basel, Gewerbestrasse 14, 4123 Allschwil, Switzerland; E-Mails: hans.deyhle@unibas.ch (H.D.); iwona.dziadowiec@gmail.com (I.D.); peter.thalmann@unibas.ch (P.T.); georg.schulz@unibas.ch (G.S.); 2 University of Applied Sciences and Arts Northwestern Switzerland FHNW, Gründenstrasse 40, 4132 Muttenz, Switzerland; E-Mail: lucy.kind@fhnw.ch

**Keywords:** enamel caries, mineralization, demineralization, self-assembling peptide, image registration, micro computed tomography, joint histogram

## Abstract

Micro computed tomography has been combined with dedicated data analysis for the *in vitro* quantification of sub-surface enamel lesion mineralization. Two artificial white spot lesions, generated on a human molar crown *in vitro*, were examined. One lesion was treated with a self-assembling peptide intended to trigger nucleation of hydroxyapatite crystals. We non-destructively determined the local X-ray attenuation within the specimens before and after treatment. The three-dimensional data was rigidly registered. Three interpolation methods, *i.e.*, nearest neighbor, tri-linear, and tri-cubic interpolation were evaluated. The mineralization of the affected regions was quantified via joint histogram analysis, *i.e.*, a voxel-by-voxel comparison of the tomography data before and after mineralization. After ten days incubation, the mean mineralization coefficient reached 35.5% for the peptide-treated specimen compared to 11.5% for the control. This pilot study does not give any evidence for the efficacy of peptide treatment nor allows estimating the necessary number of specimens to achieve significance, but shows a sound methodological approach on the basis of the joint histogram analysis.

## 1. Introduction

In caries research, the early stages of the disease are of special interest as this situation is the starting point of a comprehensive lesion. However, it is difficult to quantitatively characterize the small lesions because the methods for structural characterization, such as X-ray computed tomography, require micrometer resolution and sufficient contrast to access and segment the diseased part of the enamel. We know that the formation of dental caries is a dynamic process based on the misbalance between demineralization of enamel, *i.e.*, Ca- and P-ion loss from the hydroxyapatite prisms, and its mineralization via diffusion of the same ions from saliva back into the enamel nanostructures [1]. A detailed understanding and quantification of the involved processes will allow for deriving alternative tooth repair procedures. The caries lesions can be investigated *ex vivo* taking advantage of a wide variety of methods including microradiography [2], light microscopy [3], electron microscopy [3,4], confocal laser scanning microscopy [5], atomic force microscopy [6], and nano-indentation [7]. Some of these techniques require destructive tissue preparation or just yield a two-dimensional snapshot of the three-dimensional lesion at a particular stage during disease development. In most cases, non-destructive approaches that allow for the direct comparison of hard tissue components before and after treatment are advantageous. In this way, the kinetics of tooth mineralization become accessible [8]. The two-dimensional methods of interest include microradiography for evaluating the X-ray attenuation and the related mineral distribution across the lesion [9], as well as the scattering techniques, *i.e.*, spatially resolved, wide-angle and small-angle X-ray scattering (WAXS and SAXS), which give access to crystallite sizes, their abundance and orientation [9,10,11,12,13]. The projective nature of these methods limits the resolution in the direction parallel to the X rays to the investigated section thickness. Micro- and nano-structures in direction of the X rays are projected on the same position and cannot be discerned. Therefore, hard X-ray micro computed tomography (μCT) in absorption contrast mode is well established as a means to investigate human hard tissues with isotropic micrometer resolution [14,15,16,17]. It provides the three-dimensional distribution of the attenuation coefficient μ(*x*,*y*,*z*) that directly relates to the mineral density of human crowns. For studying comparably small mineral density differences, however, reasonable contrast is needed in addition to spatial resolution. It is established that μCT exhibits improve contrast or density resolution compared to micro-radiography, whereas the spatial resolution of the tomograms is equal at best, but generally inferior to that of radiographs [18].

The efficient application of μCT has been used to generate three-dimensional representations of calcified tissues, but despite their physical nature, these images are often only examined visually. A quantitative automatic evaluation, for example to determine the size and shape of a caries lesion or the exact spatial distribution of minerals within the lesion, proves challenging. This is due to the vast quantities of data (GB or TB), which generate time-consuming computational work with minimal projected benefits from structural and physical quantities of only a few parts of the human body. Nevertheless, μCT is a well-established technique in the dental research community [19,20].

This communication addresses the question of the extent to which μCT measurements and the subsequent quantitative data evaluation enable us to determine the mineralization of white-spot lesions that have been artificially induced according to a well-established protocol [21]. The aim is to identify alternatives to the standard caries treatments that replace affected enamel and dentin as well as the related safety zone by isotropic dental materials with their limited lifespan [22]. These conventional interventions incur significant cost and strain to the patient [23]. Consequently, the development of non-invasive, nanotechnology-based, bio-inspired approaches is essential [24,25].

For the treatment of enamel subsurface lesions, there are products such as *Curodont*™ *Repair* (Credentis AG, Windisch, Switzerland). These products involve a self-assembling β-sheet peptide that creates a supra-molecular three-dimensional, fibrous network in the acidic environment [26]. This network should trigger the nucleation of hydroxyapatite crystallites and subsequently promote the diffusion-based mineralization of the early stage carious lesions [27,28]. In order to localize the mineralization process, we applied high-resolution μCT to two artificial white spot lesions on one human molar before and after the *in vitro* treatment with *Curodont*™ *Repair* and incubation in artificial saliva. The μCT-data obtained before and after the procedure were registered to identify the changes in X-ray attenuation related to the mineral content.

## 2. Results

### 2.1. Data Interpolation

**Figure 1:**
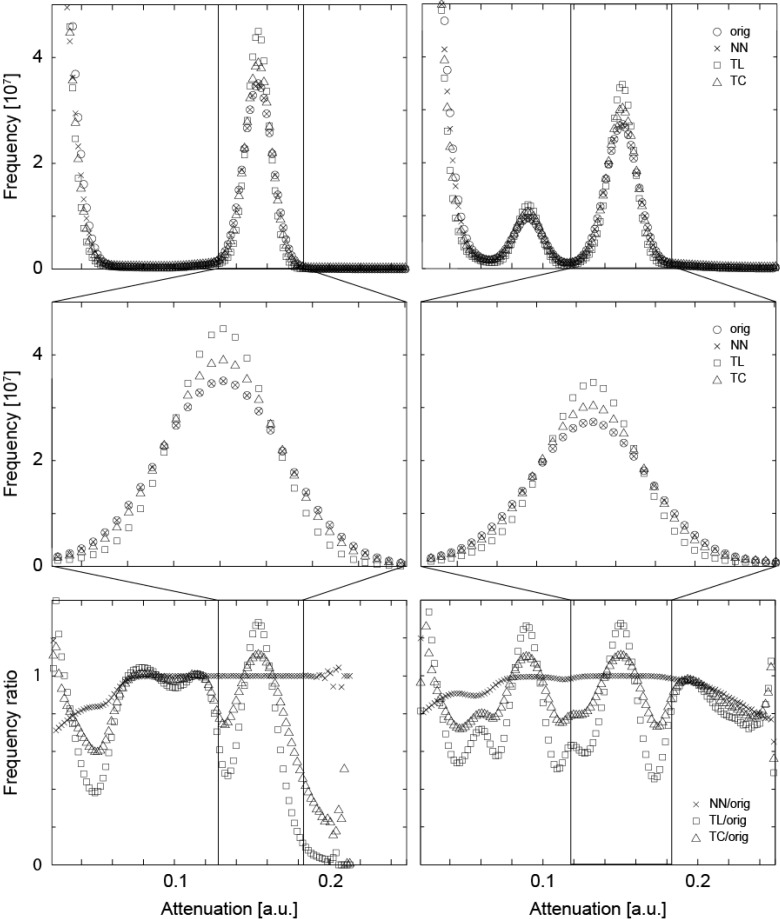
The attenuation histograms of the registered μCT datasets differ from the one of the original data. For the original data and the three interpolation methods, *i.e.*, nearest neighbor (NN), the tri-linear (TL), and the tri-cubic (TC), the histograms of the entire volumes (top) and histograms related to enamel (middle) are displayed. For the plots in the bottom row, the point-wise ratio between the histograms of the interpolated and original data was represented. For the two crown pieces (left and right column, respectively) the NN interpolation does much less influence the histogram than the TL and TC interpolation procedures. Thus, the NN algorithm is selected for the joint histogram analysis, see below.

In order to precisely match the μCT-data obtained before and after the treatment, the floating data have to be shifted and rotated within the three-dimensional space. The rotation is always associated with interpolation. Such an interpolation usually affects the noisiness of the data and the sharpness of the involved anatomical features. Linear and cubic interpolations tend to reduce noise [29], but often lead to blurring. In order to derive an appropriate interpolation for the mineralization study, we have compared three algorithms: the nearest neighbor (NN), the tri-linear (TL), and the tri-cubic (TC) interpolation, analyzing the influence on the histograms of the three-dimensional data. The diagrams in the top row of Figure 1 directly compare the histograms of the original and interpolated (rotated) data between the tissue treated with the peptide (left) and without peptide treatment (right). As we are especially interested in the changes within the lesions, the range associated with enamel is represented by the histograms in the middle row of Figure 1. TL and TC interpolations lead to a reduced peak width, caused by blurring. The NN interpolation, however, yields data closely matching the original X-ray attenuation value distribution. The bottom row of Figure 1 shows the ratio of the histograms of the interpolated data to the original ones. The NN interpolation results in ratios close to unity, whereas the TL and TC interpolations exhibit significant deviations.

### 2.2. Mineralization of the Induced Lesions

**Figure 2:**
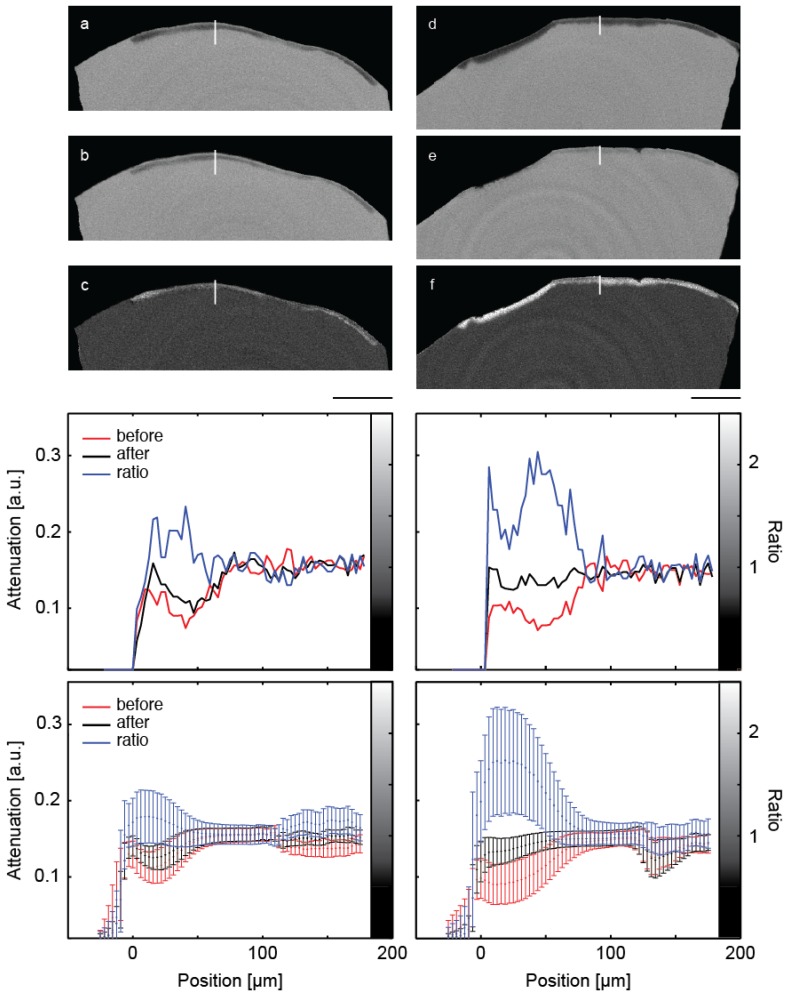
The images show selected slices of the μCT-data from the two pieces of the crown through the induced lesions. The images (**a**) and (**d**) are obtained before treatment. The related slices of the μCT-data after the treatment are images (**b**) and (**e**), (**b**) being the control and (**e**) the specimen treated with the peptide. Images (**c**) and (**f**) show the ratio of the attenuation values. The length bar corresponds to 500 μm. The diagrams in the central row show selected line plots through the lesion with locations indicated by the white lines in the images above. The increased mineral content caused by the mineralization treatments is clearly indicated. In the diagrams in the bottom row, the attenuation in the lesions is plotted against the distance from the specimen surface, integrated over the entire specimen. Error bars correspond to the standard deviation.

Figure 2 shows a representative slice through each of the μCT-data sets before and after mineralization treatment. It also contains images of the local attenuation ratios, which elucidate the increase of the mineral content as the result of the treatments. The Figure 2a,b,c belong to the control, whereas the Figure 2d,e,f show the lesion treated with the peptide.

The artificial lesions appear as dark zones below the tooth’s surface. The intact surface layer is present in both pieces of enamel. The increased attenuation resultant from the treatment is best evidenced by this ratio. In these images, the lesions appear brighter than the surrounding (Figure 2 c,f). It must be noted, however, that parts of the surface layer were lost before the second measurement.

The selected line profiles through the lesions, displayed in the central row of Figure 2, show increased X-ray attenuation after treatment in a layer up to 100 μm from the enamel surface. The application of the peptide resulted in an overall X-ray attenuation increase of about 63%, whereas a 17% increase was found for the lesion incubated without peptide. The spatial distribution from the crown surface is shown in the two diagrams of the third row in Figure 2. Here, the line plots are integrated parallel to the tooth surface and represented with their standard deviations as error bars.

### 2.3. Segmentation Using the Joint Histogram

**Figure 3:**
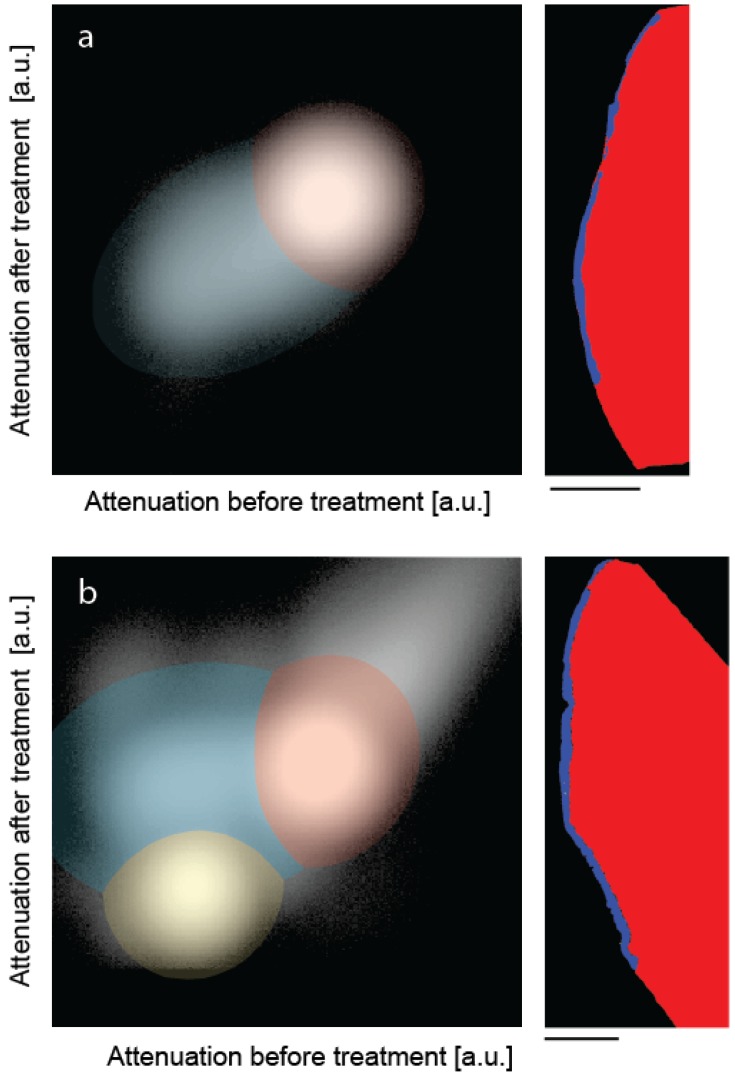
The joint histograms of the three-dimensional datasets allow segmenting the artificial lesions: (**a**) incubation of bare lesion and (**b**) incubation after peptide treatment. The selected virtual cuts through the sound enamel (red color) and the affected enamel (blue color) demonstrate the possibility of reliably segmenting the enamel lesion. The bar corresponds to the length of 500 μm.

Figure 3 shows the joint histograms of the two μCT-data sets before and after treatments [30,31]. In the present pilot study, the attenuation value of each voxel before treatment is mapped against the one after mineralization, for the control (Figure 3a) and the peptide-treated lesion (Figure 3b). If the degree of mineralization remains unaffected, the counts are distributed along the diagonal with a deviation related to the photon statistics. Mineralization gives rise to counts above the diagonal. If the treatment results in a loss of minerals, one finds the related counts below the diagonal. Artifacts such as beam hardening also lead to counts apart from the diagonal. The clusters in the joint histogram can be color-coded, as represented in Figure 3, to identify the locations of changed X-ray attenuation. The prominent clusters in Figure 3 were fitted with two-dimensional Gaussians. The intersections of the Gaussians were selected as boundaries to allow differentiating the enamel given in red, dentin represented in yellow and the artificial lesion shown in blue. Note that the dentin present in the treated specimen is not shown in the selected slice of Figure 2. No dentin was present in the control specimen. Owing to the restricted photon statistics the distributions overlap. Some of the voxels are displaced. The subsequent application of a median filter to the segmentation masks allows for the removal of such artifacts.

### 2.4. Quantification of the Mineralization

The thickness of the lesion depends on its location. The mean lesion thickness was determined to be (51 ± 16) μm with a maximum of 99 μm for the peptide-treated crown and (37 ± 11) μm with a maximum of 70 μm for the control specimen.

To quantify the effect of the incubation, both with and without peptide, we defined the mineralization coefficient *R* as:

(1) $$R=\frac{GV_{rem}-GV_{dem}}{GV_{sound}}\cdot 100\%$$

where *GV*_dem_ indicates to the mean X ray attenuation value within the lesion before incubation, *GV*_rem_ to the attenuation within the lesion after incubation, and *GV*_sound_ to the attenuation measured in unaffected enamel of the same crown.

For the control, *R* amounted to 11.5% and for the peptide-treated enamel lesion to 35.5%. Note that these are mean values over the entirety of the affected regions. It should be noted that changes in gray values between the scans might originate from beam hardening, however this is unlikely. Figure 2c,f show values close to unity with the exception of the subsurface region containing the lesion. If prominent beam hardening were present, it is also expected to manifest within the unaffected enamel. 

## 3. Discussion

The main feature of μCT is the potential to three-dimensionally and nondestructively measure the local X-ray attenuation, which is a meaningful approximation of the mineral density in bone and teeth [32,33].

To ensure the μCT-data could be compared appropriately, the pieces of the molars were measured with the same settings before and after treatment. The three-dimensional, rigid registration of the tomography data allowed for a voxel-wise direct comparison [34]. Since image registration always includes the rotation and translation of at least one dataset, resampling/interpolation is required. We have shown that the choice of the interpolation algorithm is critical. The TL and TC interpolation algorithms have a similar effect and significantly reduce the width of the peaks in the histogram (*cf.* Figure 1). Such a phenomenon might be beneficial for some visualization purposes, but in a mineralization study being able to compare data is vital. Note that smoothing, as generated through the TL and TC interpolation algorithms, is associated with a reduction in spatial resolution. The pixel size of the projection data must be small enough to display the lesion in its three-dimensional formation. In a bone study performed at a synchrotron radiation source Jorgensen *et al.* elucidated that feature reproducibility in a series of scans requires pixel sizes of less than 10% of the feature of interest [35]. This value might be specific for the setup and the tissue, but gives an indication of the required pixel size. Thus, for the μCT-setup used in the present study, the anatomical features, which change their degree of mineralization during the treatment, should be larger than 30 μm. Otherwise, only mean values can be assessed.

For the quantification of the mineralization it is crucial to identify the volume of the lesion. This is especially demanding, as the subsurface enamel lesions exhibit an inhomogeneous mineral content, and thus gradients in the X-ray attenuation. The mineral density decreases from the tooth surface towards the lesion’s center and then gradually approaches that of unaffected enamel (*cf.* Figure 2). This behavior makes simple segmentation challenging or even impossible, since one usually cannot identify any reasonable threshold. Noise due to limited photon statistics further complicates the segmentation, since the attenuation of different features with similar mineral content overlap. Region growing algorithms are better suited, but the incomplete surface layer (as seen in Figure 2) often leads to incorrect segmentation. In contrast, the segmentation on the basis of the joint histogram is straightforward and allows for the unique identification of the volumes corresponding to the enamel lesion, unaffected enamel and dentin in a similar manner as recently shown for bone and bone graft materials [30].

This study focuses on the methodological approach and relies on only two lesions. The early and preliminary results indicate that the peptides from *Curodont*™ *Repair* promote the diffusion-based mineralization from the tooth surface. Thus, our study supports recently published reports. The proposed method for the quantification of remineralization can be applied one to one to larger sample sizes.

In order to obtain the mineral content with micrometer resolution, we propose monochromatic μCT measurements as usually carried out at synchrotron radiation facilities, see for example reference [36].

## 4. Experimental Section 

### 4.1. Ethical Aspects

All procedures were conducted in accordance with the Declaration of Helsinki and according to the ethical guidelines of the Canton Basel, Switzerland. The approval of the study protocol is numbered 290/13 from the responsible Ethical Committee. The tooth was extracted for clinical reasons unrelated to the study. Written consent was given by the anonymous patient in the registration form of the Volkszahnklinik, Basel. 

### 4.2. Tissue Preparation

The root of the extracted human molar was removed. Two pieces of enamel were axially cut from the crown with a diamond band saw (Exakt Apparatebau GmbH, Nordstedt, Germany). The resulting 3 mm-thick slices had a size of 8 mm × 5 mm. Prior to demineralization the surface was covered with nail varnish leaving a 5 mm × 5 mm window.

The artificial lesions were created by incubating the specimens in an acidic demineralization buffer (50 mM acetic acid, 2.2 mM CaCl_2_, 2.2 mM NaH_2_PO_4_ titrated with 1 M KOH to the pH-value of 4.4) for three days [21]. According to the dental protocol, both specimens were pre-treated with 2% NaClO for 60 s, 35% H_3_PO_4_ for 20 s, rinsed with distilled and deionized water and dried. One lesion was incubated with 10 mg/mL P_11-4_ peptide solution acidified with 3.5% H_3_PO_4_ to promote the self assembling process [26]. The other lesion was untreated. Subsequently the two crown pieces were incubated in the mineralization buffer (1.5 mM Ca(NO_3_)_2_, 0.9 mM KH_2_PO_4_, 130 mM KCl, 60 mM Tris with HCl to pH 7.4) for a period of ten days [37].

### 4.3. Micro Computed Tomography

For the μCT-measurement, the specimens were placed in Eppendorf tubes filled with mineralization buffer. The μCT-measurements were performed with a nanotom m (phoenix|x-ray, GE Sensing & Inspection Technologies GmbH, Wunstorf, Germany). The acceleration voltage was set to 90 kVp. A filter, 0.2 mm-thin Cu, was used to increase the mean photon energy and reduce the beam-hardening artifacts. The voxel size corresponded to 3.127 μm. The 1200 radiographs with a size of 3071 × 2400 pixels were uniformly acquired over 360°. The exposure time per projection was 4.5 s. The data was reconstructed using the phoenix datos-x 2.0 reconstruction software.

### 4.4. Data Registration and Processing

To allow for a voxel-wise comparison of the tomography data acquired before and after the treatment, the datasets were rigidly registered using a registration algorithm based on the cross-correlation similarity metric [38,39] with six degrees of freedom. The μCT-data after treatment was used as reference. The rotations generally require interpolation. Three interpolation methods, nearest neighbor (NN), tri-linear (TL), and tri-cubic (TC), were compared to reach data consistency. Data processing was performed with the ITK library [40] and self-written MATLAB^®^ code (Matlab2013b, The MathWorks, Inc., Natick, MA, USA).

## 5. Conclusions

The three-dimensional registration of the μCT data before and after the saline incubation requires interpolation. The NN interpolation algorithm fits well, whereas the TL and TC algorithms significantly alter the attenuation histogram. Once reasonable three-dimensional registration of μCT data before and after incubation has been obtained, the joint histogram allows segmenting healthy enamel, dentin, and the subsurface lesion. More importantly, the mineralization of the lesion can be identified and subsequently quantified in detail.

This preliminary mineralization study with two artificial caries lesions focused on methodology indicates that the peptides from *Curodont*™ *Repair* may promote the diffusion-based mineralization. A possible protocol for the identification and standardized evaluation of mineralized lesions, which takes advantage of well-registered μCT-data, was developed. The present pilot study, however, does not give any evidence for the efficacy of the treatment procedure. It also does not allow for estimating the necessary number of samples to reach significance. Consequently, this communication is considered a starting point for a sound mineralization study and as a methodology-oriented paper.
